# Rapid contraction of the anterior capsule after phacoemulsification in uveitis: A case report

**DOI:** 10.1097/MD.0000000000043129

**Published:** 2025-07-11

**Authors:** Xiaojing Liu, Jie Yang, Yuhan Zhen, Xinran Zhai, Xin Zhang, Yanhui Xu, Yue Zhang, Zhimin Chen

**Affiliations:** a Department of Ophthalmology Teaching and Researching, Hebei Medical University, Hebei, People’s Republic of China; b Department of Cataract, Hebei Eye Hospital, Hebei Provincial Key Laboratory of Ophthalmology, Hebei Provincial Clinical Research Center for Eye Diseases, Hebei, People’s Republic of China.

**Keywords:** capsular contraction syndrome, continuous curvilinear capsulorhexis, intraocular lens, phacoemulsification, uveitis

## Abstract

**Rationale:**

While capsular contraction syndrome (CCS) typically develops months after cataract surgery, its potential for rapid progression in uveitis patients remains poorly characterized. This case addresses the critical knowledge gap regarding ultra–early-onset CCS and its diagnostic challenges in inflammatory eye disease.

**Patient concerns:**

A 63-year-old male with controlled uveitis reported rapidly worsening blurred vision (15 days postphacoemulsification) despite initially good postoperative visual acuity (0.4 at 1 week). The vision deteriorated to hand motion at 10 cm, unresponsive to anti-inflammatory treatment.

**Diagnoses:**

Right eye CCS, right eye posterior uveitis, and left eye senile cataract.

**Interventions:**

Initial failed Nd:YAG laser capsulotomy due to extreme fibrosis, 2-day nonsteroidal anti-inflammatory drug pretreatment, and capsular sac relaxing surgery combined with intraocular lens alignment surgery.

**Outcomes:**

Immediate intraoperative restoration of capsular opening, best-corrected visual acuity recovered to 0.5.

**Lessons:**

This fastest-reported CCS case (15-day onset) demonstrates: uveitis patients require CCS surveillance within 2 to 4 weeks postoperatively; rapid vision decline without active inflammation should prompt CCS consideration; and surgical intervention remains definitive for advanced fibrosis cases.

## 1. Introduction

Capsular contraction syndrome (CCS) is a rare but difficult to diagnose complication after cataract surgery, characterized by unexplained contraction of the intraocular lens (IOL) capsule between 3 and 30 weeks after cataract surgery, leading to visual problems such as glare, loss of vision, etc. The diagnostic difficulty lies in the non-specificity of the symptoms and the tendency to confuse them with other ocular problems, which, together with the low prevalence, makes clinical recognition and management particularly challenging. In this case report, we describe a rare case of CCS that occurred within a very short period of time after surgery and was treated surgically with a good recovery.

## 2. Case report

The patient was a 63-year-old man with a 5-year history of uveitis in the right eye. He underwent cataract surgery on the right eye 1 month ago, with hydrophilic IOL implantation and postoperative visual acuity of 0.6. He developed blurred vision in the right eye for 15 days with visual acuity of 10 cm index and was treated conservatively for 1 week at a traditional Chinese medicine ophthalmology department, but the loss of visual acuity worsened. Therefore, the patient was referred to our department on October 21, 2024 for further treatment.

**Figure 1.**
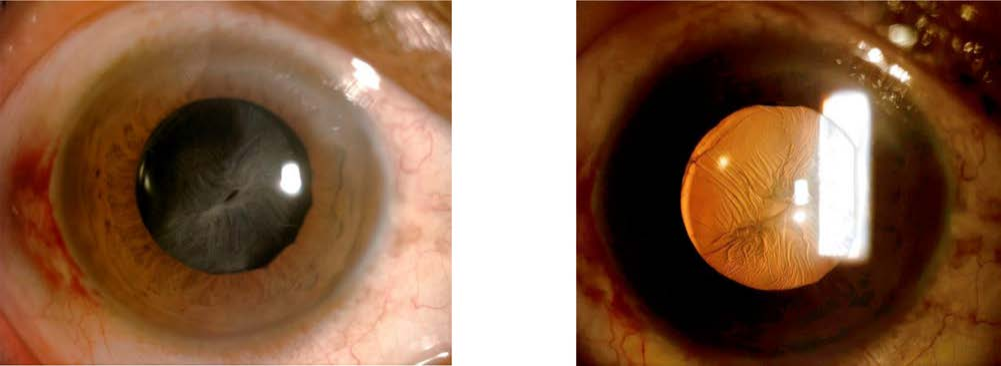
Anterior segment showing significant shrinkage and fibrosis of the anterior lens capsule membrane, which is lacunar and approximately 1 mm in diameter.

**Figure 2.**
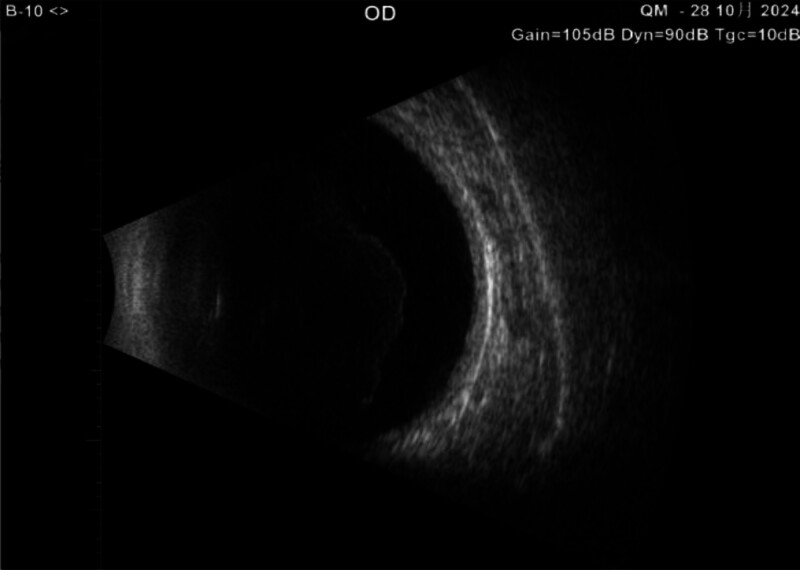
Ophthalmologic ultrasound: vitreous opacity in the right eye and posterior vitreous detachment in the right eye.

**Figure 3.**
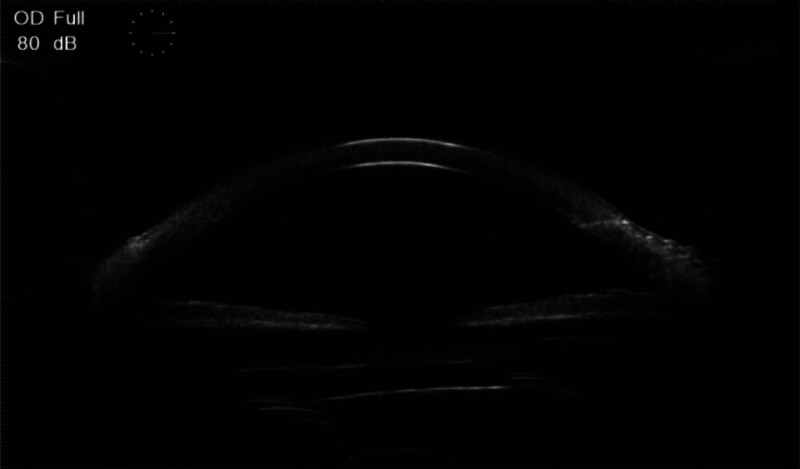
UBM examination showed dense turbidity of the anterior capsule membrane of the IOL, and the IOL was located within the capsular bag.

**Figure 4.**
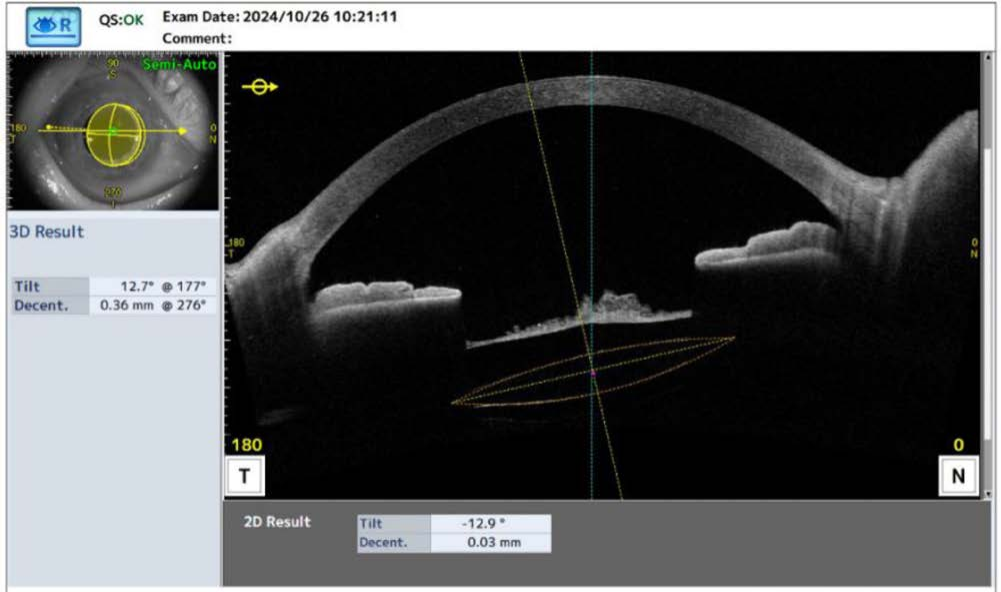
OCT examination showed dense turbidity of the anterior capsule membrane of the IOL, and the IOL was located within the capsular bag.

**Figure 5.**
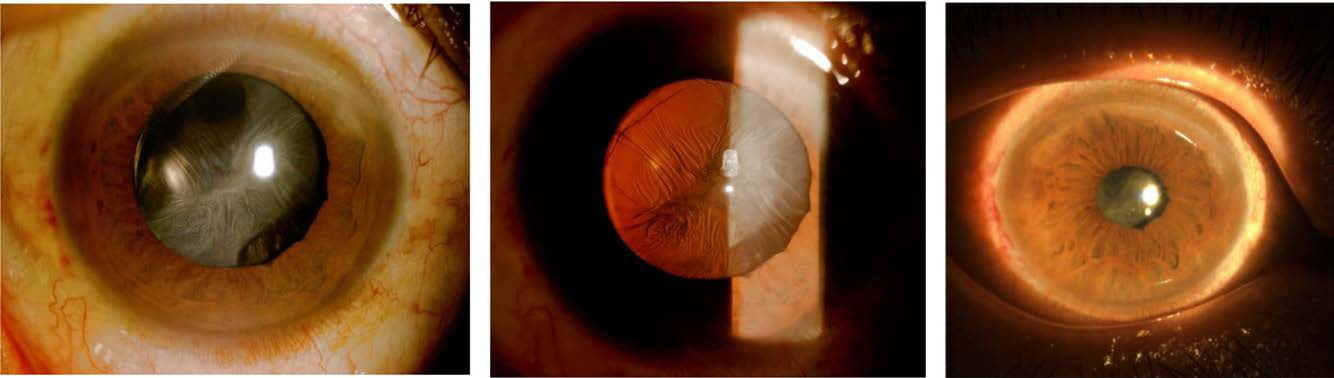
Anterior segment photograph showing anterior capsular contraction causing a total occlusion of the visual axis.

Ophthalmologic examination: right eye visual acuity 10 cm index, left eye visual acuity 0.4. Intraocular pressure was normal bilaterally. Right eye: conjunctiva slightly congested, cornea transparent, anterior chamber deep, pupil diameter about 3 mm, lens anterior capsule membrane visibly thickened and wrinkled, capsule membrane fibrosis white dense opacity, anterior capsule membrane residual tearing, capsule mouth tilted to the nasal side, was slit-like, diameter approximately 1 mm, the IOL is in the capsular bag is pulled off position, the vitreous body is blurred to see the large number of grayish-white flocculation opacities, the remaining structures are not visible (Fig. 1). The left eye had lens cortical clouding, the rest was not abnormal. Ophthalmologic ultrasound: vitreous opacity in the right eye and posterior vitreous detachment in the right eye (Fig. 2). Both ocular UBM and anterior segment OCT examinations showed dense turbidity of the anterior capsule membrane of the IOL, and the IOL was located within the capsular bag (Figs. 3 and 4). The diagnosis is as follows: right eye CCS; right eye posterior uveitis; left eye senile cataract. YAG laser incision treatment was tried but was unsuccessful because the turbidity of the capsule was too high and dense for the laser to effectively penetrate or incise the capsule. Therefore, the patient was prepared for surgery and given NSAIDs for 2 days preoperatively for anti-inflammatory treatment. On the day of surgery, the patient’s anterior lens capsule opening was completely occluded and mechanized (Fig. 5).

**Figure 6.**
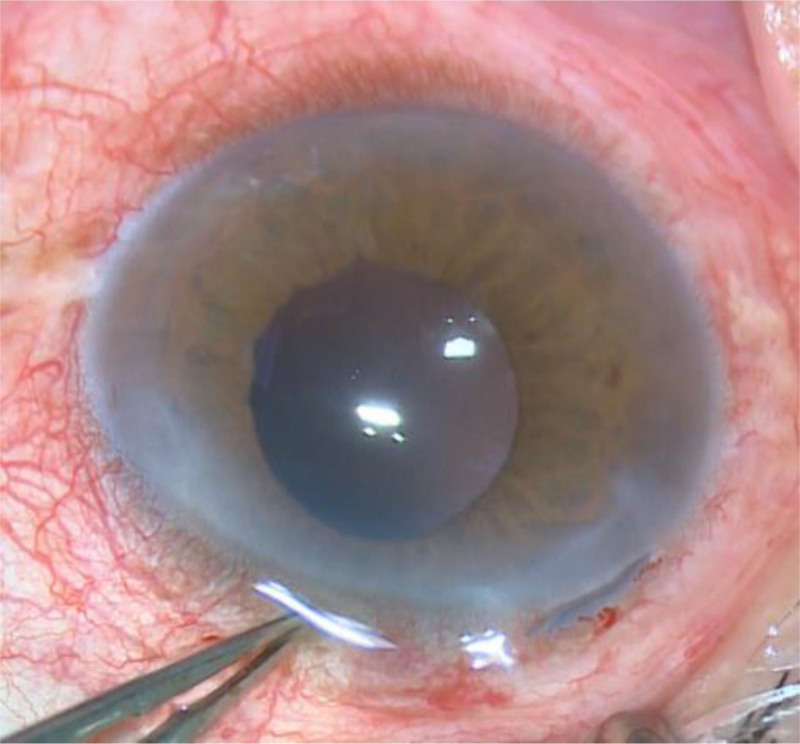
After releasing the cystic sac.

Finally, the patient underwent a right eye capsular sac relaxing surgery combined with IOL alignment surgery (Fig. 6). The surgical procedure was successful, and the postoperative visual acuity in the right eye was 0.5 with an intraocular pressure of 11 mm Hg.

## 3. Discussion

CCS is a syndrome characterized by a reduction in the diameter of the equatorial portion of the lens capsule, accompanied by fibrosis of the anterior lens capsule and a reduction in the area of the tearing capsule region, caused by various reasons after intracapsular implantation of IOL.^[1,2]^ Clinical manifestations include reduction in the size of the anterior capsule opening area, narrowing of the capsule contraction, mechanical opacification of the residual lens capsule, IOL deviation, reduced visual acuity and glare. It is a complication of continuous circular capsulotomy (CCC) in cataract surgery with an incidence of approximately 1.4% to 14%, usually occurring 3 to 30 weeks postoperatively and slowing down the contraction of the capsular bag after 3 months. The presence of CCS tends to cause tilt, eccentricity or twisted deformation of the IOL loops in the capsular bag, or even dislocation, which can lead to vision loss, glare, refractive error, etc., and reduce the patient’s postoperative visual quality. The pathophysiology is not fully understood. It is thought that residual lens epithelial cells from the anterior capsule proliferate, migrate and transform into myofibroblasts, resulting in this newly formed fibrous membrane.^[3]^ The degree of anterior capsular bag constriction also depends on a number of individual factors, particularly the quality of the patient’s own ligaments, the state of the lens capsule, systemic and ocular pathologies, and surgical complications.^[4,5]^

Our case has 2 unusual features: firstly, CCS occurred within a very short time after surgery, and secondly, it proved to be very intense, leading to complete occlusion of the optic axis and severe visual impairment within a short period of time. A number of risk factors for CCS have been reported in the literature, such as high myopia, retinitis pigmentosa, pseudoexfoliation syndrome, glaucoma, uveitis, and diabetes suspensory ligament fragility or intraocular inflammation.^[4,6]^ Our patient had one of these features, uveitis. As a high-risk disease for capsular bag contraction, uveitis itself causes an inflammatory response and disruption of the blood-aqueous barrier, which are important factors leading to capsular bag contraction. On the other hand, the weak zonular structure in uveitis eyes exacerbates the imbalance between the centripetal force of the anterior capsule opening margin (fibrotic change) and the centrifugal force of the capsular zonules, making them susceptible to the risk of CCS after cataract surgery. We think that a relatively smaller capsulorhexis facilitated the anterior capsule contraction in the affected eye. Bisevac et al^[7]^ determined that during the pathogenesis of uveitis, inflammatory cells may produce various cytokines, such as transforming growth factor β, through paracrine secretion and other means, which further stimulate myofibroblast activity and collagen synthesis, leading to capsular bag contraction. At the same time, the immune response may also lead to damage and contraction of the lens capsular bag. In addition, the occurrence of capsular bag contraction is not only related to the above-mentioned diseases, but may also be influenced by a variety of factors such as surgical manipulation, IOL material and design, and postoperative inflammatory response.^[8]^

In addition to a history of uveitis, we believe that the IOL used in this case may be one of the predisposing factors for CCS. Hydrophilic material IOLs are designed to be easily compressed to fit through microincisions, and these IOLs are also soft and thin. The inherent flexibility makes the Rayner IOL more susceptible to deformation when CCS occurs. Secondly, the hydrophilic surface is a favorable environment for cell proliferation and migration, which tends to lead to proliferation and migration of lens epithelial cells; therefore, the capsular bag is slightly less biocompatible, with a higher incidence of turbidity in the anterior and posterior capsular membranes. Thus, all of the above characteristics of the IOL used in our case may have resulted in less resistance to capsular contraction.

To date, according to our search of the PubMED and MEDLINE databases, there are no published case reports regarding contraction of the anterior capsule following cataract surgery in patients with uveitis. In most of the previous reports, CCC occurred mostly months to years after cataract surgery, and only a few other primary cases presented with problems similar to our patient less than a month after surgery. Examples include high myopia, retinitis pigmentosa, and pseudoexfoliation syndrome. Jin-Poi et al^[9]^ and Nikpoor^[10]^ et al report a case similar to ours. Their patients, all with retinitis pigmentosa, all experienced rapid contraction of the anterior capsule over a 2 to 3 week postoperative period and were treated with the YAG laser, and the patients had satisfactory postoperative visual acuity. Also, both our case and the cases of Jin-Poi et al and Nikpoor and Stone showed signs of IOL instability in the capsular bag, which was promptly treated with early diagnosis and intervention. It is presumed that the etiology of anterior capsule contraction in retinitis pigmentosa patients involves an imbalance between the centrifugal forces of the zonules and the forces of the intraocular lens haptics, and the centripetal forces of the proliferative and metaplastic residual lens epithelial cells.^[1]^ Eung et al^[11]^ reported a patient with pseudoexfoliation syndrome and a 1-piece hydrophilic acrylic square rim IOL implanted in the right eye, which resulted in CCC 2 months later and a clear visual axis by Nd:YAG laser dissolution of the anterior membrane. They attributed the occurrence of CCS to an imbalance between centripetal and centrifugal forces that act on the zonules and the capsulorhexis edge, smaller CCC and failure to completely clean the lens epithelial cell intraoperatively. The cases mentioned above, although of different primary diseases, are similar to our case in that the rapid onset of postoperative CCS was suspected to be due to an abnormality in the patient’s own capsule ligament. This was the focus of our attention.

Treatment of CCS varies depending on the degree of visual disturbance, the stability of capsule and IOL, and the co-existence of other complications.^[12]^ In the face of CCS, anterior segment OCT swept-source imaging helps to differentiate the anterior from the posterior capsule and to assess CSS thickness. It also helps analyze lens position, fibrosis thickness, and the distance between the fibrosis and the IOL. These parameters are key in deciding the treatment approach.^[3]^ Most clinicians use Nd:YAG laser anterior capsulotomy as a first-line treatment for CCS by creating radial incisions through photodisruption.^[13]^ However, the amount of laser energy delivered may weaken the zonular fiber, break the posterior capsule or destabilize the IOL, and it should therefore be carefully performed.^[14]^ In this patient, complete occlusion of the capsular bag opening as well as capsular bag thickening impeded the success of prelaser capsulotomy, thus requiring surgical capsulotomy and IOL adjustment. Therefore, we recommend fundus examination and close follow-up of patients at high risk for CCS, and once a trend toward progressive atrophy of the anterior capsule is detected during follow-up, laser anterior capsulotomy should be performed promptly to avoid further complications.

This case highlights the importance of considering techniques to prevent or minimize capsular contracture after cataract surgery in patients with uveitis. Several intraoperative measures to reduce the incidence of CCS have been proposed in the literature in the past. Specifically: A 5.5-mm capsulorhexis which covers the IOL’s optic seems to prevent epithelial cells proliferation, creating a so-called barrier effect; anterior capsular polishing, capsular tension ring and acrylic 3-piece IOL implantation seem to effectively hinder this complication. We suggest that, without changing these techniques, clinicians should be aware of possible complications in patients with uveitis and closely monitor these developments to reduce the likelihood of reoperation.

## 4. Conclusion

Rapid anterior capsule contraction is a rare condition, but it is important to emphasize to your ophthalmologist the need to prevent anterior capsule contraction in patients with uveitis. Larger capsulorhexis size, use of a capsular tension ring, proper selection of IOL, careful capsular lens epithelial cell clean-up, and radial relaxing incisions in the anterior lens capsule, have all been advocated as effective measures to prevent or limit the occurrence of anterior capsule contraction. Therefore, patients should undergo a thorough ocular and systemic evaluation prior to cataract surgery to assess surgical risks and develop an individualized surgical plan. Postoperative ocular conditions should also be closely monitored, and timely measures should be taken to prevent and treat complications such as capsular bag contraction.

## Author contributions

**Data curation:** Xinran Zhai.

**Investigation:** Yuhan Zhen, Xin Zhang.

**Methodology:** Zhimin Chen.

**Supervision:** Yanhui Xu, Yue Zhang.

**Writing – original draft:** Xiaojing Liu.

**Writing – review & editing:** Jie Yang, Zhimin Chen.
